# Multiple Electrolyte and Metabolic Emergencies in a Single Patient

**DOI:** 10.1155/2017/4521319

**Published:** 2017-01-31

**Authors:** Caprice Cadacio, Phuong-Thu Pham, Ruchika Bhasin, Anita Kamarzarian, Phuong-Chi Pham

**Affiliations:** ^1^Olive View-UCLA Medical Center, 14445 Olive View Drive, 2B-182, Sylmar, CA 91342, USA; ^2^Ronald Reagan UCLA Medical Center, 200 Medical Plaza, Los Angeles, CA 90095, USA

## Abstract

While some electrolyte disturbances are immediately life-threatening and must be emergently treated, others may be delayed without immediate adverse consequences. We discuss a patient with alcoholism and diabetes mellitus type 2 who presented with volume depletion and multiple life-threatening electrolyte and metabolic derangements including severe hyponatremia (serum sodium concentration [S_Na_] 107 mEq/L), hypophosphatemia (“undetectable,” <1.0 mg/dL), and hypokalemia (2.2 mEq/L), moderate diabetic ketoacidosis ([DKA], pH 7.21, serum anion gap [S_AG_] 37) and hypocalcemia (ionized calcium 4.0 mg/dL), mild hypomagnesemia (1.6 mg/dL), and electrocardiogram with prolonged QTc. Following two liters of normal saline and associated increase in S_Na_ by 4 mEq/L and serum osmolality by 2.4 mosm/Kg, renal service was consulted. We were challenged with minimizing the correction of S_Na_ (or effective serum osmolality) to avoid the osmotic demyelinating syndrome while replacing volume, potassium, phosphorus, calcium, and magnesium and concurrently treating DKA. Our management plan was further complicated by an episode of significant aquaresis. A stepwise approach was strategized to prioritize and correct all disturbances with considerations that the treatment of one condition could affect or directly worsen another. The current case demonstrates that a thorough understanding of electrolyte physiology is required in managing complex electrolyte disturbances to avoid disastrous outcomes.

## 1. Introduction

Managing various electrolyte and metabolic disturbances is generally a simple task for nephrologist. However, in complex cases, one must be vigilant of potentially life-threatening interactions among multiple simultaneous treatment plans and cautiously formulate a comprehensive treatment algorithm to prevent disastrous outcomes.

## 2. Case Report

*Clinical History*. A 33-year old male with known alcohol abuse and diabetes mellitus type 2 presented with a two-day history of nausea, vomiting, watery diarrhea, and light headedness. Patient denied fevers and chills but endorsed mild midepigastric dull pain and poor oral intake.

*Physical Exam*. Temperature was 37.2°C, blood pressure 114/85 mmHg, heart rate 109 beats per minute, respiratory rate 22 per minute, and oxygen saturation 98%. Patient was acutely ill-appearing, slow in verbal responses, alert and oriented, and free of stigmata of advanced liver disease. Oral mucosa was dry. Heart exam was notable for tachycardia. Lungs were clear bilaterally. Abdomen had hypoactive bowel sounds and mild midepigastric tenderness without guarding or rebound. Extremities were significant for a few ecchymoses. Neurological exam was nonfocal.

*Initial Laboratory Data*. Serum chemistries at presentation and hospital course are presented in Table 1. Most notable abnormalities included serum sodium (S_Na_) 107 mEq/L, potassium (S_K_) 2.8 mEq/L, total CO_2_ 12 mEq/L, glucose 331 mg/dL, and anion gap (S_AG_) 37 mEq/L.* Others* were mild transaminitis, mildly elevated lipase, and hemoglobin 12 g/dL.

Renal service was consulted following the increase in S_Na_ from 107 to 111 mEq/L over one hour (effective serum osmolality [S_osm_] increase of 2.4 mosm/Kg) with the administration of two liters of normal saline.

*Additional Investigations*. Renal service requested STAT serum phosphorus and magnesium which resulted as <1 mg/dL and 1.6 mg/dL, respectively.* Other findings* were moderate serum ketones, lactic acid 1 mmol/L, and S_osm_ 255 mosm/Kg (serum osmolality gap 6 mosm/Kg).

*Venous Blood Gas at Six Hours following Presentation to Emergency Department (ED)*. pH was 7.40, pCO_2_ 17 mmHg, and HCO_3_ 10 mEq/L (S_AG_ 31 mEq/L), and* at thirteen hours*, pH was 7.21, pCO_2_ 14 mmHg, and HCO_3_ 6 mEq/L (S_AG_ 30 mEq/L).* Urine studies* show osmolality 450 mosm/Kg, sodium 25 mEq/L, and potassium 25 mEq/L.

*Diagnoses*. Diagnoses included volume depletion, diabetic ketoacidosis (DKA) (with initial concurrent metabolic and respiratory alkaloses), severe hyponatremia, hypokalemia, hypophosphatemia, mild to moderate hypocalcemia, and mild hypomagnesemia.

*Clinical Follow-Up*. Patient received emergent potassium chloride (KCl) infusion via a central line (200 mL/hr of 100 mEq/L KCl solution [total 480 mEq KCl]) and potassium phosphate (KPO_4_) (total 240 mmol), magnesium sulfate (total 8 g), and calcium gluconate (total 4 g) via peripheral lines. Oral thiamine and folate were given daily. All net fluid and effective solutes (sodium and potassium) were closely monitored. Calculations were performed (based on CurbsideConsultant.com) every six hours to readjust all fluid rates as needed to ensure a goal sodium correction rate of 4–6 mEq/L/24 hours. Treatment of DKA was intentionally delayed until S_K_ reached 2.9 mEq/L to avoid insulin-driven intracellular potassium uptake, exacerbation of hypokalemia, and precipitation of life-threatening arrhythmias. On hospital day 3, patient developed significant aquaresis with approximated free water clearance of 230 to 300 mL/hr (urine output of 3140 mL over 8 hour; urine studies: osmolality 186 mosm/Kg, sodium 13 mEq/L and potassium 16 mEq/L; S_Na_ 122 mEq/L). Two micrograms of desmopressin (DDAVP) and two liters of electrolyte-free water were given intravenously to slow urine output and prevent rapid overcorrection of S_Na_, respectively. Over the first four hospital days, S_Na_ corrected at an average of 5 mEq/L/day. Additionally, patient also underwent upper gastroendoscopy for gastrointestinal bleed and nausea which revealed diffuse gastritis, presumed to be induced by his chronic alcohol consumption. His nausea resolved with proton pump inhibitor and supportive care.

## 3. Discussion

**Figure 1:**
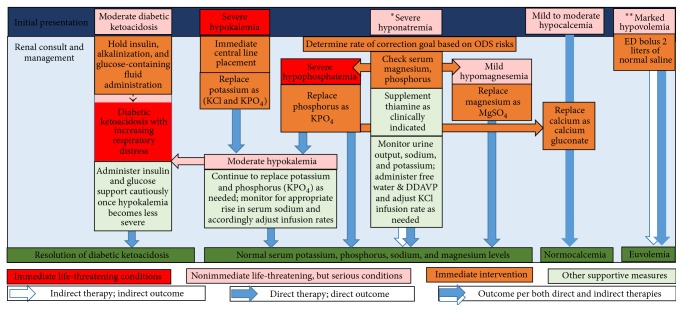
Algorithm for the treatment of multiple concurrent life-threatening disturbances. ^*∗*^For hyponatremia, correction resulted from both potassium infusion (indirect therapy) and fine adjustment with intermittent free water infusion and single administration of desmopressin (direct therapy) to achieve rate of correction goal during an episode of aquaresis. ^*∗∗*^For volume depletion, patient received two liters of normal saline on presentation to the emergency department (direct therapy) and continuous KCl infusion at 200 mL/hour (indirect therapy, i.e., the main purpose for KCl infusion, was potassium replacement, but patient benefited from the infusion as maintenance intravenous fluid) over the following 2 to 3 days while his oral intake was poor. ODS: osmotic demyelination syndrome; ED: emergency department; DDAVP: desmopressin.

The current case was challenged by multiple concurrent problems including the need for continuing volume support and substantial administration of both KCl and KPO_4_ without rapidly correcting hyponatremia, optimization of all treatable osmotic demyelinating syndrome (ODS) risks, correction of DKA to prevent respiratory decompensation without worsening the life-threatening hypokalemia, and intermittent infusions of magnesium sulfate and calcium gluconate while anticipating and managing any significant aquaresis without derailing the planned S_Na_ correction rate. The algorithm for the comprehensive management of current patient is summarized in Figure 1.

*Hypokalemia* was the most life-threatening and one of first abnormalities to be treated emergently. Etiologies likely included poor dietary intake, renal wasting given recent vomiting and poorly controlled diabetes, and diarrhea. Immediate life-saving interventions included KCl infusion via a central line along with instructions to avoid alkalinization or administration of insulin or glucose-containing fluids, the latter because of endogenous insulin secretion, and to prevent intracellular K^+^-shift and worsening hypokalemia.

While aggressive potassium administration was critical, S_Na_ level had to be closely monitored because potassium effectively increases S_Na_. Serum sodium concentration has been shown to be directly proportional to the sum of total exchangeable Na^+^ and K^+^ content [1]. Mechanisms whereby K^+^ administration can raise S_Na_ include the following [2]:

1. intracellular K^+^-uptake induces an equivalent extracellular Na^+^-movement and hence increased S_Na_,
2. parallel K^+^-Cl- intracellular uptake leads to increased intracellular osmolality which leads to intracellular free water shift and lower extracellular free water volume and hence increased S_Na_, or
3. intracellular K^+^-uptake induces an equivalent extracellular H^+^-movement to maintain electrical neutrality. While H^+^ can bind to the extracellular buffer system and not perturb extracellular osmolality, the intracellular K^+^-gained increases intracellular osmolality and hence intracellular free water shift. The lower extracellular free water volume increases extracellular S_Na_.

Given the direct effect of K^+^ on S_Na_, both sources of potassium, KCl and KPO_4_, were accounted for in all calculations for expected changes in S_Na_. Further sodium administration was withheld because potassium supplement alone was determined to be sufficient to correct hyponatremia. Failure to recognize this fact and unwarranted infusion of sodium-containing solutions could have easily led to rapid hyponatremia overcorrection.

*Hypophosphatemia* may be a risk factor for ODS [3]. Our routine hyponatremia treatment protocol requested a STAT level, which was likely life-saving. Patient's severe hypophosphatemia could arise from poor oral intake, renal wasting, hypomagnesemia-induced skeletal resistance to parathyroid hormone (PTH 161 pg/mL, 1,25 (OH)_2_ vitamin D 146 pg/mL), and possibly some degree of intracellular uptake associated with primary respiratory alkalosis at presentation [4]. The latter could induce intracellular alkalemia and associated increased glycolysis and intracellular uptake of phosphorus for ATP production [5]. Phosphorus replacement was given emergently to avoid respiratory and cardiac arrest among other potential serious complications.

*Volume depletion* is typically managed with normal saline (NS), but not in current case. Patient received predominantly K^+^-containing fluids (200 mL/hour of 100 mEq/L KCl solution and 25 to 50 mL/hour of KPO_4_ solution [15 mmol KPO_4_ mixed in 200 mL of either normal saline or sterile water as indicated by S_Na_]). Since K^+^ is an effective solute, either K^+^ or Na^+^-containing solutions that are relatively isotonic to patient's effective osmolality will effectively expand intravascular volume. With the exception of two liters of NS given in the ED, patient's total body volume was repleted and maintained with the infusion of K^+^-containing fluids intended for potassium and phosphorus repletion. Failure to recognize the volume expansion capacity of relatively isotonic KCl-containing fluid and unwarranted infusion of NS for the sole purpose of volume support would have complicated the treatment of hyponatremia. Additionally, high volume infusions of multiple fluids would have led to excess urinary loss of ketone bodies necessary for bicarbonate production with insulin administration [6].

*Hypocalcemia* was likely due to poor nutrition, malabsorption, and hypomagnesemia-induced hypoparathyroidism [7, 8]. Given prolonged QTc, patient received low dose calcium gluconate intravenously following the initiation of KPO_4_ administration to avoid any potential calcium-induced worsening of severe hypophosphatemia via calcium phosphate precipitation.

*Diabetic ketoacidosis* was potentially life-threatening, but not the most serious derangement. Insulin administration was intentionally delayed to avoid worsening of hypokalemia. Patient's respiratory status, however, was closely monitored. Once S_K_ reached 2.9 mEq/L, insulin was cautiously given to avoid respiratory failure as patient exerted high work of breathing to compensate for the metabolic acidosis. Within 12 hours of insulin administration, S_AG_, likely all reflecting ketone bodies, decreased from 30 mEq/L to 19 mEq/L with a parallel increase in total CO_2_ from 6 to 14 mEq/L. The rapid inverse change in S_AG_ and total CO_2_ demonstrates perfectly how* sufficient* fluid resuscitation,* not excessive* fluid administration with resultant urinary loss of serum ketone bodies, can allow for preservation of serum ketone bodies, where rapid hepatic conversion to bicarbonate occurs with insulin administration [6]. Patient's respiratory status also improved significantly with correction of metabolic acidosis.

*Hyponatremia* was likely multifactorial and includes continuing free water intake in the presence of enhanced secretion of antidiuretic hormone (ADH) with volume depletion and/or inappropriate ADH secretion in the setting of nausea, “beer potomania,” and small degree of hyperglycemia-induced extracellular free water shift. The major goal in hyponatremia correction is ODS prevention. This requires both setting an appropriate correction goal and recognizing and optimizing any concurrent factors that could potentiate the risk of developing ODS [3, 9]. The correction goal was determined to be 4 to a maximum of 6 mEq/L/day because of patient's ODS risks including severe hyponatremia, hypokalemia, alcoholism, hypophosphatemia, hypomagnesemia, glucose intolerance, and presumed thiamine deficiency [3, 9]. Routine assessment of reversible ODS risk factors is warranted because electrolytes such as phosphorus and magnesium are not routinely measured at many institutions, including our own (Table 2).

In terms of actual hyponatremia correction, the infusion of potassium-containing solutions alone was sufficient. Patient's S_Na_ improved daily at expected rates from the predominant infusions of KCl and KPO_4_ solutions (Table 1). Additionally, as per our routine hyponatremia management protocol, monitoring of urine output, sodium, and potassium was done at regular intervals. Patient indeed developed a significant aquaretic phase when electrolyte-free water and DDAVP were promptly given to divert hyponatremia overcorrection. Significant aquaresis during the treatment of hyponatremia may occur in multiple clinical settings and generally stems from the rapid cessation of ADH secretion following the correction of underlying stimuli that induced ADH secretion like correction of volume depletion, nausea, pain, among others [10]. In current case, the aquaretic phase was likely due to the correction of volume depletion and nausea.

*Hypomagnesemia* was likely due to poor oral intake, gastrointestinal malabsorption, and possibly urinary loss associated with diabetes mellitus [11]. Patient was monitored closely for hypomagnesemia and treated as needed.

*Thiamine* was also supplemented given history of alcoholism to minimize ODS risk [4].

*Respiratory and metabolic alkalosis* on presentation were likely due to pain/anxiety and volume depletion, respectively. Both conditions resolved with comprehensive supportive care.

## 4. Conclusions

We present a complex case involving multiple life-threatening electrolyte and metabolic disturbances which demonstrates the critical need for prioritization for the treatment of each abnormality and considerations for all interactions among multiple concurrent treatment plans.

Aggressive potassium replacement prior to the administration of insulin for the DKA is vital to prevent worsening of life-threatening hypokalemia.

Both sodium and potassium are equivalent effective solutes. Hyponatremia can be corrected with the predominant infusion of potassium. Similarly, volume expansion with relatively isotonic KCl solution is as effective as NaCl in current case of severe hypokalemia.

The treatment of hyponatremia must incorporate correction rate, monitoring, and treatment of all factors (potassium, phosphorus, magnesium, glucose, and thiamine) associated with increased ODS risks. Additionally, during the treatment of hyponatremia, transient aquaresis may arise for various reasons and must be anticipated and immediately treated to avoid rapid overcorrection [11].

Insulin effectively converts ketone bodies to bicarbonate if the former have not been lost in the urine with excessive fluid administration.

Despite multiple life-threatening electrolyte and metabolic disturbances, patient was discharged within twelve days in good condition and continued to do well at one month follow-up.

Teaching points are summarized in Table 2.

## Figures and Tables

**Table 1:** Clinical data.

Serum chemistry	Admission	After 2 L normal saline (renal service was consulted)	12 to 14 hours after admission	8 hours after insulin administration (30 to 32 hours after admission)	Discharge (12 days after admission)	Total amount replaced during hospitalization	Electrolyte concentrations of fluids administered
Sodium (mEq/L)	107	111	113	117	132	2 liters of normal saline at presentation	154 mEq/L

Potassium (mEq/L)	2.8	**2.2**	2.9 to 3.2	**2.7**	4.5	480 mEq KCl	100 mEq/L

Total CO_2_ (mEq/L)	12	11	6	14	25	Corrected with insulin	

Serum anion gap (mEq/L)	37	31	30	19	10	Corrected with insulin	—

Glucose (mg/dL)	331	248	250	108	217	Corrected with insulin	—

Phosphorus (mg/dL)	Not done	<**1.0**	<**1.0**	<**1.0**	5.5	240 mmol KPO_4_	15 mmol mixed in 200 mL normal saline or free water as needed to achieve sodium correction goal

Total calcium (mg/dL)	7.2	6.9	**6.8**	6.8	8.4	4 g calcium gluconate (9.3 mmol)	10% solution
Ionized calcium (mg/dL) [normal 4.6–5.4]	4.1	Not done	**4.0**	Not done	Not done		

Magnesium (mg/dL)	**1.6**	Not done	3.1	Not done	1.7	8 g	4 g mixed in 250 mL normal saline

**Table 2:** Teaching points box.

General teaching points	Comments pertinent to current case
Treatment of one electrolyte or metabolic abnormality can critically worsen another. In a patient with multiple disturbances, a comprehensive management plan must *prioritize* the most to least life-threatening disturbance and treat accordingly. Additionally, consideration must be made for all possible *treatment interactions*, particularly when the treatment of a less critical problem can exacerbate a life-threatening condition	(i) Hypokalemia and hypophosphatemia were the two most life-threatening conditions in current patient. Since the treatment of diabetic ketoacidosis (DKA) with insulin with or without glucose support could have exacerbated the severe hypokalemia and precipitate cardiac arrest, such treatment was intentionally delayed. Aggressive potassium replacement with both KCl and KPO_4_ to achieve a safer serum potassium level was done PRIOR to the treatment of DKA

*Comprehensive* protocol for the management of hyponatremia: (i) Determine osmotic demyelination risks (ODS) and appropriate rate of correction (ii) Assess and treat all correctable ODS risks (hypokalemia, hypomagnesemia, hypophosphatemia, altered glucose metabolism, and clinical need for thiamine) (iii) Understand that potassium can increase serum sodium exactly as if the same amount of sodium is being administered (iv) Monitor urine output and its content of sodium and potassium for any possible aquaretic phase	(i) While hyponatremia was being corrected with KCl and KPO_4_ infusions, the immediate plan to monitor and correct factors [hypophosphatemia and hypomagnesemia] associated with high ODS risks led to the prompt recognition of severe and life-threatening hypophosphatemia (ii) In addition to the correction of concurrent electrolyte disturbances, thiamine supplementation should be considered in patients with malnutrition or alcoholism as thiamine deficiency has been implicated in increasing the risk for ODS (iii) Current patient's serum sodium concentration improved as planned with only potassium-containing fluids (iv) Aggressive monitoring of both urine output and content of effective electrolytes (sodium and potassium) allows for prompt intervention and thus prevention of rapid over correction of hyponatremia

KCl is as effective as NaCl solution as a volume expander and may be preferred or even required when potassium is critically deficient	(i) The substitution of KCl for NaCl solution for volume expansion can only be given in cases of severe hypokalemia. The rate and concentration of the KCl solution MUST be adjusted to assure a safe rate of increase in serum sodium (ii) Note that a maximum of 20 mEq of KCl may be continuously infused per hour through a central venous catheter

Respiratory hyperventilation and metabolic acidosis associated with diabetic ketoacidosis alone may be easily and promptly reversed with the administration of insulin. Persistent abnormalities should thus prompt an evaluation for other underlying etiologies	(i) Following the administration of insulin, our patient's respiratory status improved from a respiratory rate up to mid-30's breaths per minute down to mid-20's within 24 hours (ii) Similarly, patient's serum total CO_2_ improved from 6 to 14 mEq/L within 8 hours (iii) Note, however, close monitoring of potassium must be done following insulin and glucose support therapy. With the initiation of insulin therapy, patient's serum potassium decreased from a high of 3.2 mEq/L to 2.7 mEq/L within 7 hours

## References

[1] Edelman I. S., Leibman J., O'Meara M. P., Birkenfeld L. W. (1958). Interrelations between serum sodium concentration, serum osmolarity and total exchangeable sodium, total exchangeable potassium and total body water. *The Journal of Clinical Investigation*.

[2] Rose B. D. (1994). *Clinical Physiology of Acid-Base and Electrolyte Disorders*.

[3] Pham P.-M. T., Pham P.-A. T., Pham S. V., Pham P.-T. T., Pham P.-T. T., Pham P.-C. T. (2015). Correction of hyponatremia and osmotic demyelinating syndrome: have we neglected to think intracellularly?. *Clinical and Experimental Nephrology*.

[4] Freitag J. J., Martin K. J., Conrades M. B. (1979). Evidence for skeletal resistance to parathyroid hormone in magnesium deficiency. Studies in isolated perfused bone. *Journal of Clinical Investigation*.

[5] Brautbar N., Leibovici H., Massry S. G. (1983). On the mechanism of hypophosphatemia during acute hyperventilation: evidence for increased muscle glycolysis. *Mineral and Electrolyte Metabolism*.

[6] Felig P. (1974). Diabetic ketoacidosis. *New England Journal of Medicine*.

[7] Chase L. R., Slatopolsky E. (1974). Secretion and metabolic efficacy of parathyroid hormone in patients with severe hypomagnesemia. *Journal of Clinical Endocrinology and Metabolism*.

[8] Rude R. K., Oldham S. B., Singer F. R. (1976). Functional hypoparathyroidism and parathyroid hormone end‐organ resistance in human magnesium deficiency. *Clinical Endocrinology*.

[9] Verbalis J. G., Goldsmith S. R., Greenberg A. (2013). Diagnosis, evaluation, and treatment of hyponatremia: expert panel recommendations. *The American Journal of Medicine*.

[10] Pham P. C., Chen P. V., Pham P. T. (2000). Overcorrection of hyponatremia: where do we go wrong?. *American Journal of Kidney Diseases*.

[11] Pham P.-C. T., Pham P.-M. T., Pham S. V., Miller J. M., Pham P.-T. T. (2007). Hypomagnesemia in patients with type 2 diabetes. *Clinical Journal of the American Society of Nephrology*.

